# Nanocomposites of LLDPE and Surface-Modified Cellulose Nanocrystals Prepared by Melt Processing

**DOI:** 10.3390/molecules23071782

**Published:** 2018-07-19

**Authors:** Alojz Anžlovar, Matjaž Kunaver, Andraž Krajnc, Ema Žagar

**Affiliations:** 1 Department of Polymer Chemistry and Technology, National Institute of Chemistry, Hajdrihova 19, SI-1000 Ljubljana, Slovenia; matjaz.kunaver@ki.si; 2 Department of Inorganic Chemistry and Technology, National Institute of Chemistry, Hajdrihova 19, SI-1000 Ljubljana, Slovenia; andraz.krajnc@ki.si

**Keywords:** cellulose nanocrystals, surface modification, esterification, nanocomposites, linear low-density polyethylene, critical percolation concentration

## Abstract

Cellulose nanocrystals (CNCs) were surface modified by esterification in tetrahydrofuran (THF) at 25 °C using different catalysts and anhydrides bearing different alkyl side chain lengths. Unmodified and acetic anhydride (AcAnh)-modified CNCs were studied as potential nanofillers for linear low-density poly(ethylene) (LLDPE). Nanocomposites were prepared by melt processing. Determination of the size and size distribution of CNCs in the nanocomposites by SEM revealed an enhanced compatibility of the AcAnh-modified CNCs with the LLDPE matrix, since the average size of the aggregates of the modified CNCs (0.5–5 μm) was smaller compared to that of the unmodified CNCs (2–20 μm). Tensile test experiments revealed an increase in the nanocomposites’ stiffness and strain at break—by 20% and up to 90%, respectively—at the CNC concentration of 5 wt %, which is close to the critical percolation concentration. Since the CNC nanofiller simultaneously reduced LLDPE crystallinity, the reinforcement effect of CNCs was hampered. Therefore, the molding temperature was increased to 120 °C, and, in this way, the greatest increase of the Young’s modulus was achieved (by ~45%). Despite the enhanced compatibility of the AcAnh-modified CNCs with the LLDPE matrix, no additional effect on the mechanical properties of the nanocomposites was observed in comparison to the unmodified CNC.

## 1. Introduction

Recently, cellulose nanocrystals (CNCs) have received significant attention from the scientific community, since they are distinguished by their renewability, biodegradability, biocompatibility, and excellent mechanical properties. CNCs can be prepared from various cellulose sources, most frequently via an acid hydrolysis process, most often using sulfuric acid and seldom other inorganic acids (hydrochloric or phosphoric acid) [1]. The formation of CNCs is ascribed to selective hydrolysis of the amorphous cellulose regions as a consequence of their faster degradation, compared to the crystalline regions [2]. Impressive mechanical properties make CNC a highly attractive reinforcing nanofiller for various polymer matrices due to the high specific Young’s modulus of CNC (a ratio between the Young’s modulus and density; ~85 J g^−1^), which is even higher than that of the steel (~25 J g^−1^) [3].

In order to enhance the range of CNC application, they are often chemically modified to change their surface characteristics [4]. Namely, CNCs are rather polar structures due to a large number of hydroxyl groups on the cellulose surface, as well as the presence of additional sulfonic groups if the hydrolysis is performed by the sulfuric acid. Therefore, the CNC surface energy characteristics are tuned if they are intended to be used as a nanofiller in nonpolar/hydrophobic polymer matrices, with the aim to improve the compatibility between the nanocomposite’s constituents [5]. The main challenge in the chemical modification of CNCs is to conduct the process at the surface only, while preserving the cellulose’s original morphology and the crystal integrity. The destruction of the crystalline domains results in impaired CNC mechanical properties, and consequently, in deteriorated CNC reinforcement of the polymer matrices [5,6].

Literature has reported on various chemical modifications of the CNC surface [6,7]. Some chemical reactions, such as sulfonation with sulfuric acid [8] and carboxylation with ammonium persulfate [9], can be performed during the CNC isolation process, however, in most cases the chemical modifications are performed after CNC isolation [10], e.g., esterification [11], etherification [12], oxidation [13], silylation [14], or urethanization [15]. Chemical modification of CNCs can be carried out in one-step or in two-step reactions, e.g., amidation [16] and various types of click reactions (azide–alkyne [17] or thiol-ene [18]). Moreover, polymer grafting reactions onto [19] or from the CNC surface [20] have been intensively studied.

One of the most studied chemical modifications of cellulose is the esterification of its hydroxyl groups. In general, esterification reactions can be divided into five groups with respect to the reagent and catalyst used; i.e., acid halides, acid anhydrides, acid catalyzed reaction with carboxylic acids, in situ activated carboxylic acids, and transesterification reaction [21]. Acetylation (methyl ester formation) with acetic anhydride, a commonly used acetylating agent, is frequently used for chemical modification of the cellulose fibers, microfibrillated cellulose [22], and nanofibrillated cellulose [23]. One possible method of esterifying the nanofibrillated cellulose is through hydrolysis and subsequent Fischer esterification [24]. Another approach includes a transesterification reaction using, for example, vinyl acetate as a reagent [25]. An alternative method to esterify the cellulose hydroxyl groups using less reactive fatty acids is the so-called mixed esterification, in which acetic anhydride is used as a co-reactant [26]. An innovative approach to cellulose esterification includes the use of a lipase enzyme as a catalyst [27]. Citric acid was also studied as a catalyst for cellulose acetylation [28]. Alternatively, CNCs can be esterified with glutaric anhydride in an ionic liquid medium [29].

One of the most studied polymer matrices which were reinforced by CNCs is poly(ethylene) (PE) [30]. Mokhena and Luyt [31] studied the reinforcing effect of silane-modified CNCs on the mechanical properties of high-density PE (HDPE) and low-density PE (LDPE). They reported an improvement of the tensile modulus and a decrease in the elongation at break, however, the stress at break was improved solely in the case of HDPE-based nanocomposites. de Menezes et al. [32] studied composites of LDPE and CNCs modified with long alkyl chains, which were prepared by melt processing. They observed a significant decrease in the elongation at break, while the tensile strength and Young’s modulus remained practically unchanged. Li et al. [33] studied composites of HDPE reinforced by either unmodified or with PEG-modified CNCs, which were prepared by extrusion. The authors reported on the improved bending strength and modulus when the CNCs and HDPE were premixed in water suspension and freeze dried.

Herein, we disclose an optimized procedure for esterification of the CNC hydroxyl groups with the anhydrides of different alkyl chain lengths. The purpose of is to modify the surface of cellulose for further application of modified CNCs; that is, as a reinforcing nanofiller in a linear low-density poly(ethylene) (LLDPE) matrix, thus preparing LLDPE/CNC nanocomposites with improved properties by melt processing.

## 2. Results and Discussion

### 2.1. Modification of CNCs

**Figure 1:**
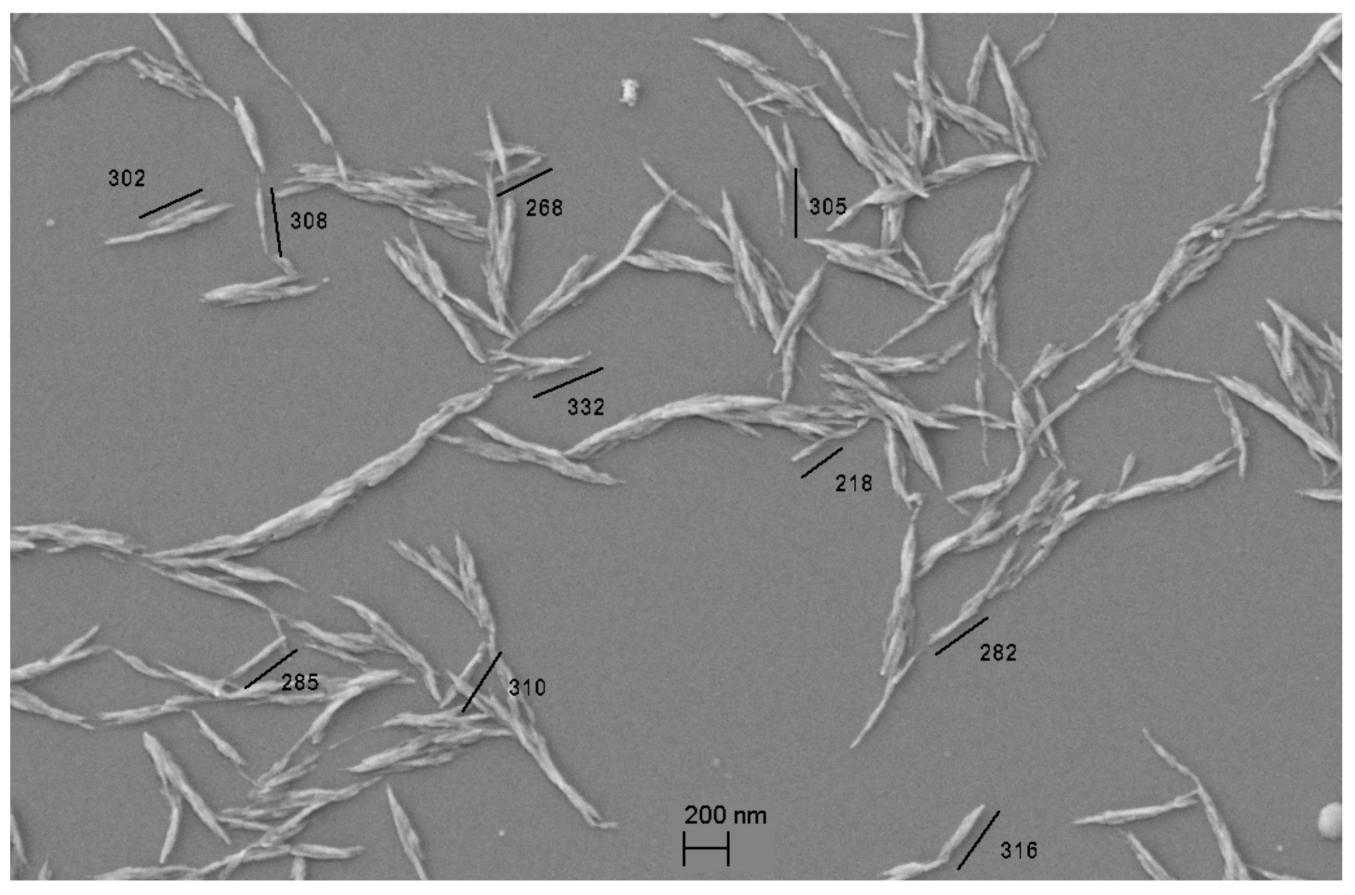
SEM micrography of the cellulose nanocrystals isolated by a ‘polyol method’.

CNCs were prepared using cotton linters as a raw material by the previously reported ‘polyol method’ in a diethylene glycol/glycerol mixture (70/30) using methane sulfonic acid as a catalyst (Figure 1) [34,35,36]. CNCs have an average particle length of 291 ± 24 nm and diameter ranging from 20 nm to 50 nm, as determined from SEM micrography (Figure 1).

**Figure 2:**
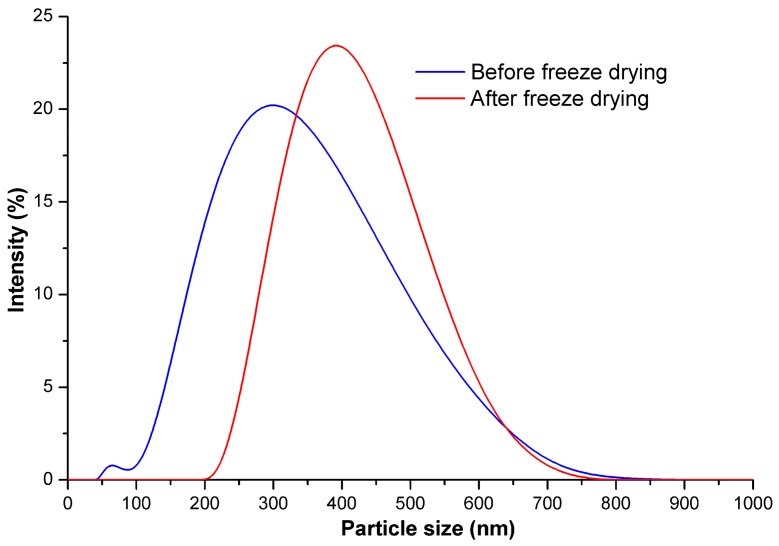
Cellulose nanocrystal (CNC) particle-size distributions before and after freeze drying. Dynamic light-scattering (DLS) measurements were performed in DI H_2_O for the never-dried and the freeze-dried CNC samples.

CNCs can form extremely stable and ordered structures due to the strong interparticle hydrogen bonding between the surface hydroxyl groups. To minimize particle aggregation, CNCs are usually used in the form of the so-called never-dried dispersion. However, in this case, the dry CNC content was not clearly defined, and therefore, a stoichiometry of the modification reaction is questionable. In an alternative approach, the dry CNC particles are used in the form of cellulose aerogel that is obtained by freeze-drying CNCs. During freeze-drying, the original CNC structure in water is mostly preserved, and CNC aggregation is, to a high extent, prevented. Such nanocellulose can be redispersed in various media using sonotrode sonication, as was already reported in [23]. Compared to the original CNCs, the average particle size and particle size distribution of the freeze-dried CNCs are somewhat larger and narrower, respectively, as indicated by dynamic light-scattering (DLS) (Figure 2). Such results are ascribed to partial aggregation and rearrangement of the smallest CNC particles into the larger structures during freeze drying.

**Scheme 1:**
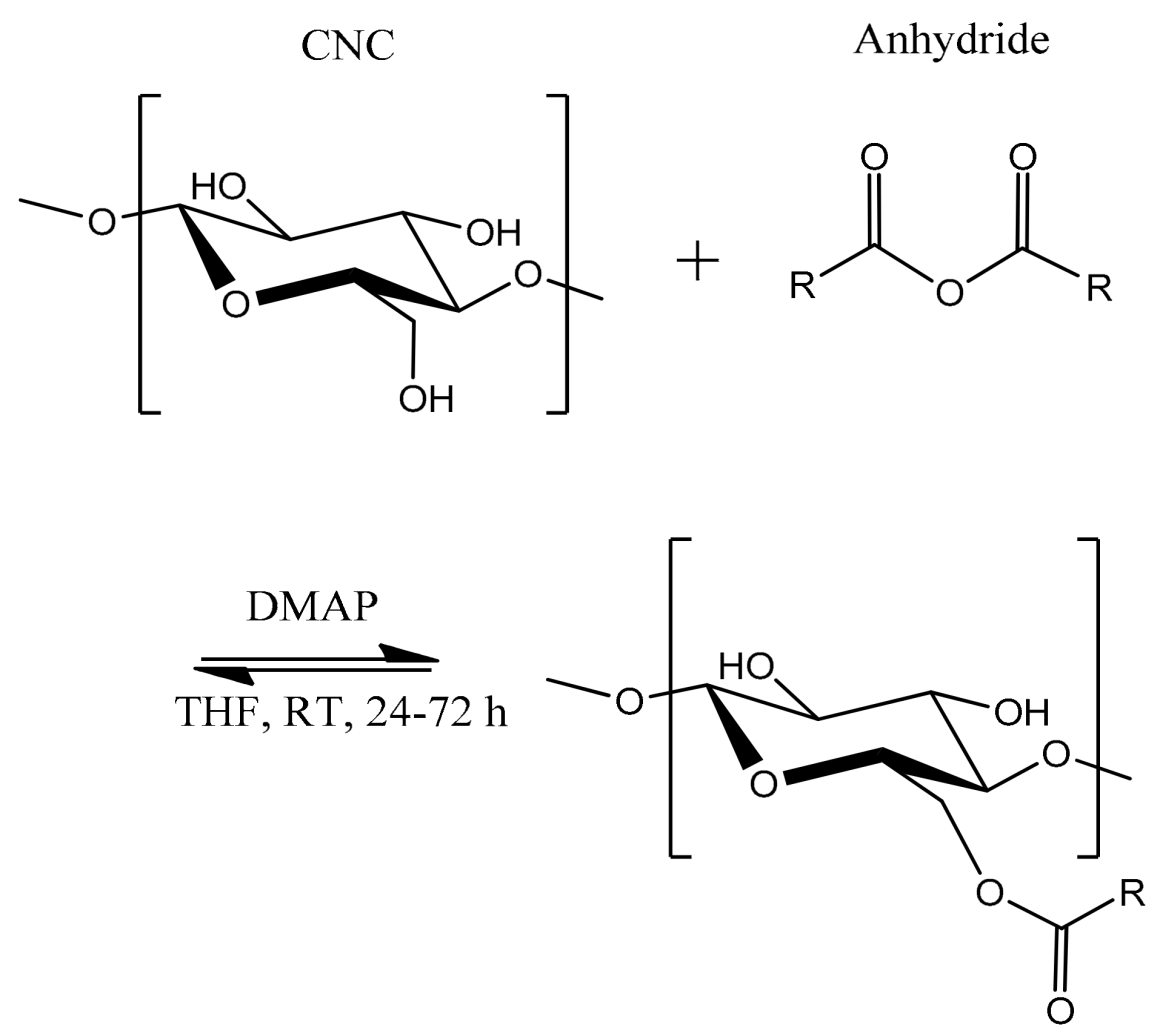
Reaction scheme of CNC esterification reaction with various anhydrides.

Surface modification is challenging, since the CNC crystalline structure should be preserved without dissolution of the modified cellulose chains in the reaction medium. Modification reactions of CNCs have to take place at the interface between the solid and liquid phases, limiting the modification to only a few chemical reactions, which usually demand highly reactive modifying agents. In our case, the hydroxyl groups on the CNC surface were esterified by various anhydrides (Scheme 1). For this purpose, we used a synthetic procedure developed for the esterification of various organic compounds, including poorly reactive alcohols, with the anhydrides at low temperatures and in high yields [37].

**Figure 3:**
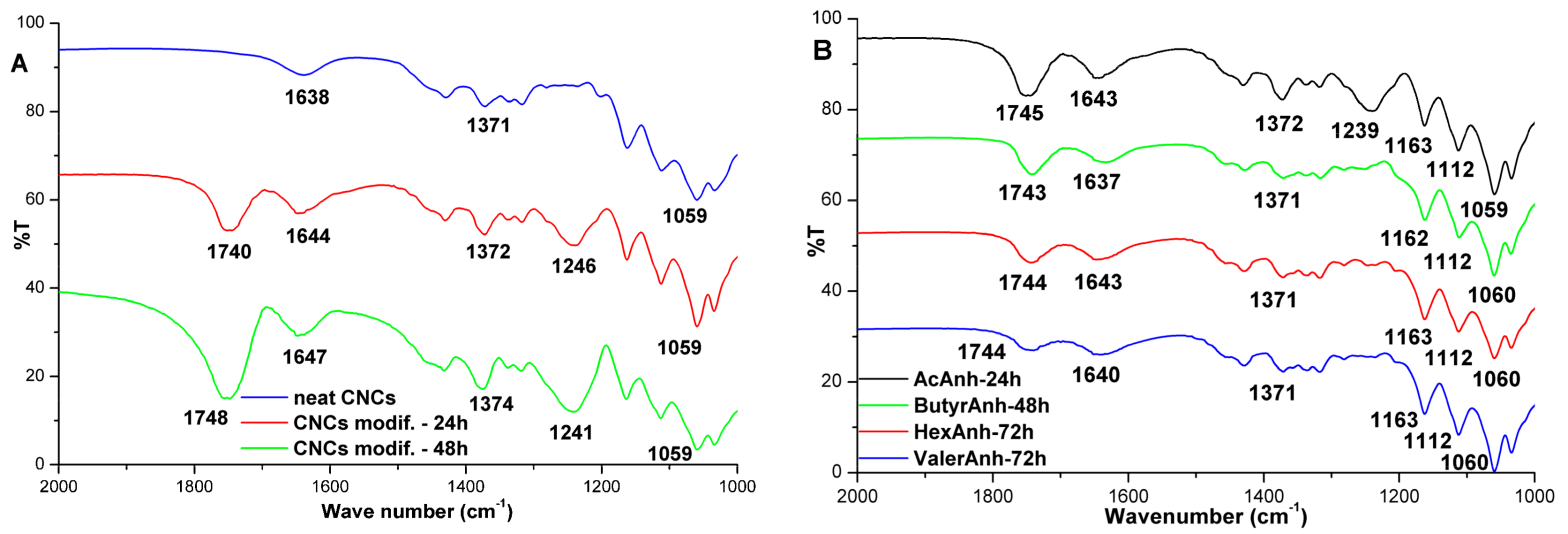
(**A**) FTIR spectra of CNCs modified with acetic anhydride (AcAnh) at room temperature and different reaction times; (**B**) FTIR spectra of CNCs modified with anhydrides of various alkyl chain lengths.

**Figure 4:**
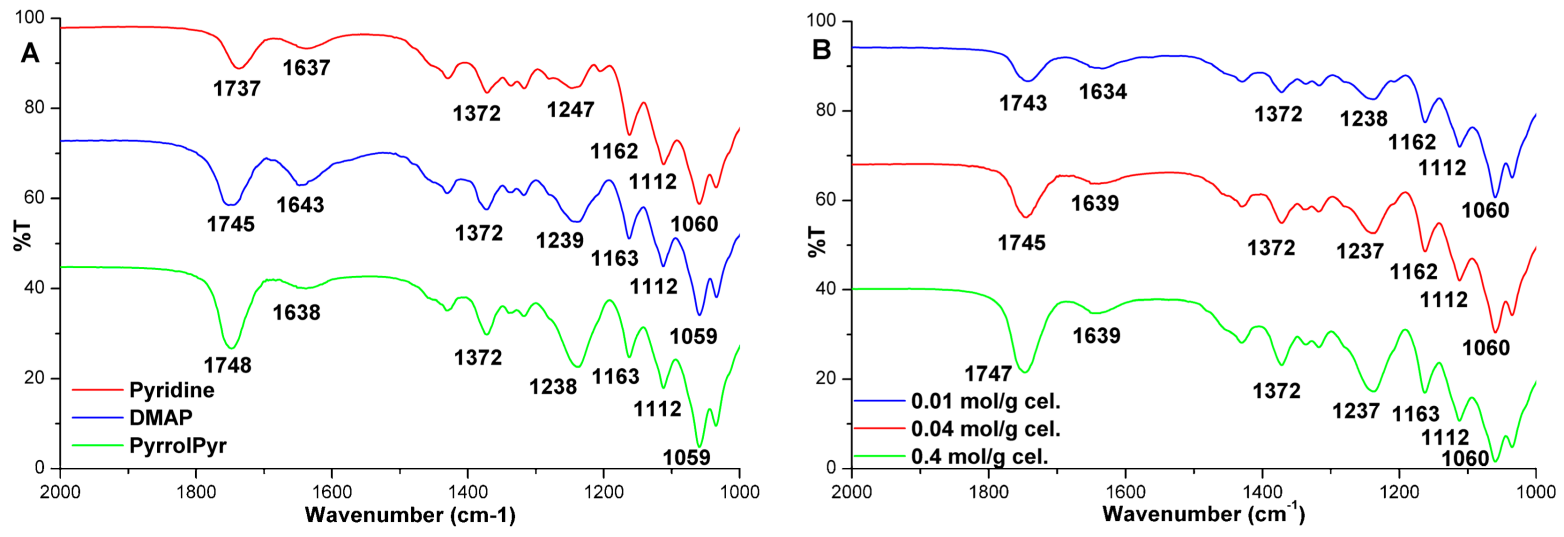
(**A**) FTIR spectra of CNCs modified with AcAnh as a function of the catalyst used; (**B**) FTIR spectra of CNCs modified with AcAnh as a function of the AcAnh/cellulose ratio.

**Figure 5:**
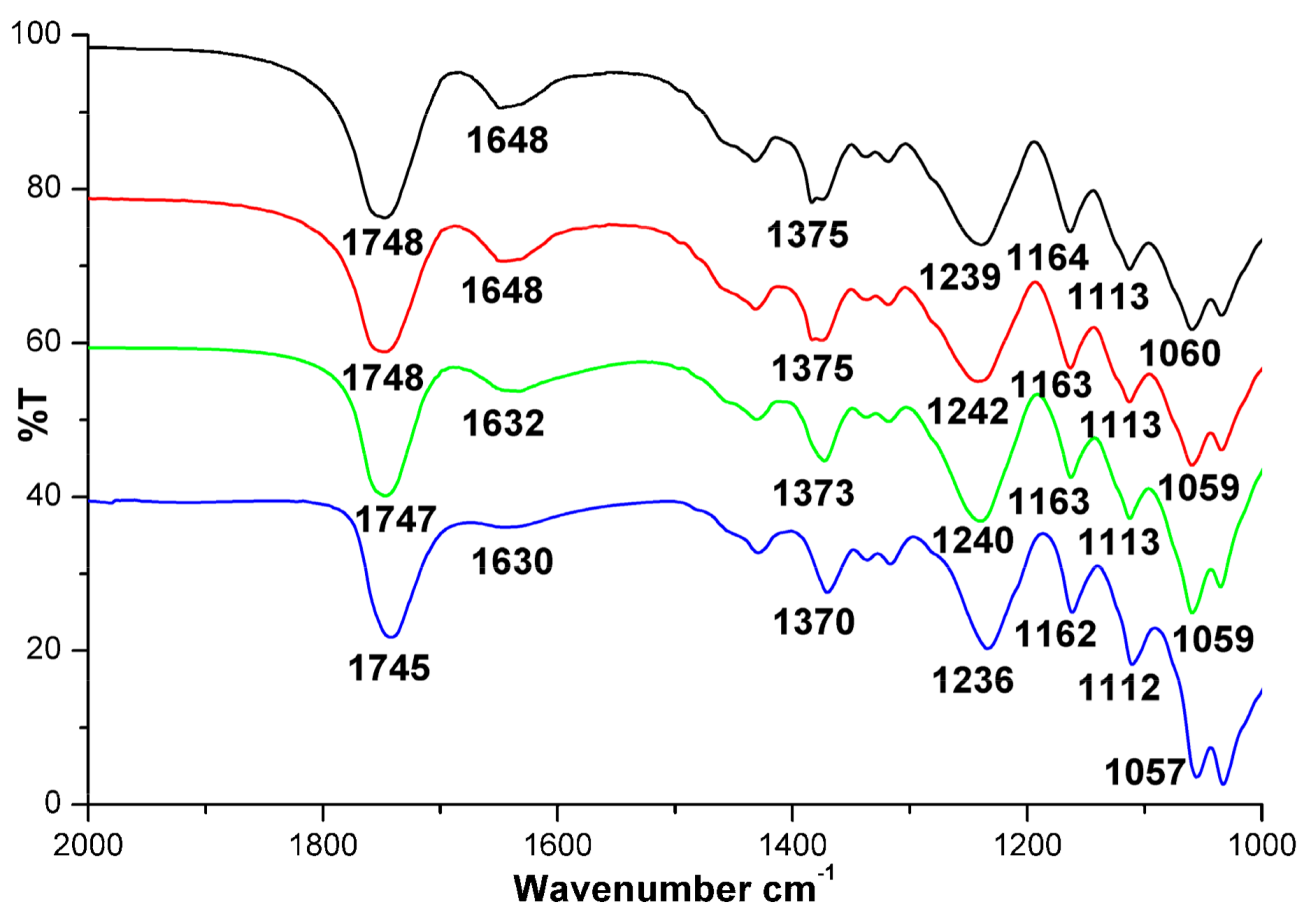
FTIR spectra of CNCs modified with AcAnh; the reproducibility test.

FTIR spectra of CNCs modified with various anhydrides reveal a typical ester carbonyl absorption band positioned between 1720 cm^−1^ and 1750 cm^−1^, depending on the type of the anhydride used, as well as an additional absorption band at approx. 1240 cm^−1^, which is characteristic of the aliphatic ester C–O stretching vibration (Figure 3, Figure 4 and Figure 5). The absorption band at ~1640 cm^−1^ is due to the H–O–H bending vibration of the absorbed water. The absorption bands at 1431 cm^−1^ and 1372 cm^−1^ are associated with the CH_3_, CH_2_, and CH stretching and bending vibrations, while the bands at 1163 cm^−1^, 1112 cm^−1^, and 1060 cm^−1^ are due to C–O–C bridge stretching vibration and the asymmetric pyranose ring stretching vibration.

The anhydrides applied to the CNC modification were acetic (AcAnh), butyric (ButyrAnh), hexanoic (HexAnh), and valeric (ValerAnh). The reaction time needed for successful esterification depended on the anhydride type; 24 h was necessary for AcAnh (Figure 3A) and 48 h for ButyrAnh, while the anhydrides bearing longer aliphatic chains—HexAnh and ValerAnh—required a longer reaction time of 72 h (Figure 3B). The increased intensity of the absorption band associated with the carbonyl stretching vibration between 1720 cm^−1^ and 1750 cm^−1^ indicates higher yields of the esterification reaction as a consequence of longer reaction times (Figure 3A). The lower reactivity of the anhydrides with longer side chains (Figure 3B), the efficiency of the selected catalysts (Figure 4A), and the influence of different AcAnh/cellulose ratios (Figure 4B) on the degree of the esterification reaction can be inferred from the intensity of the carbonyl stretching vibration. These results reveal that the highest reactivity is for AcAnh, while the lower reactivity of the other anhydrides is attributed to a sterically hindered reaction due to the presence of longer alkyl chains in their structure. The esterification reaction was catalyzed by three different catalysts, i.e., pyridine, 4-dimethylaminopyridine (DMAP), and pyrrolidinopyridine (PyrrolPyr). The highest catalytic activity was observed for PyrrolPyr, followed by DMAP, while pyridine was found to be least effective (Figure 4A). For further experiments, we chose DMAP as a catalyst due to its low price, and AcAnh as the most reactive reactant and for which we expected to have the least impact on the formation of the CNC percolation network during nanocomposite preparation. In further experiments, the quantity of AcAnh relative to the cellulose mass was optimized. It was found that 0.4 mol of AcAnh per 1 g of CNCs is the most appropriate (Figure 4B). Moreover, the esterification reaction of CNCs with anhydrides was proved to be reproducible, as shown by the FTIR spectra of the CNC samples prepared in four parallels (Figure 5). For experiments discussed hereafter, the following standard reaction conditions were used: room temperature, reaction time of 24 h, 0.4 mol of AcAnh per 1 g of dry CNC, and 3.2 mmol of DMAP per 1 g of dry CNC.

**Figure 6:**
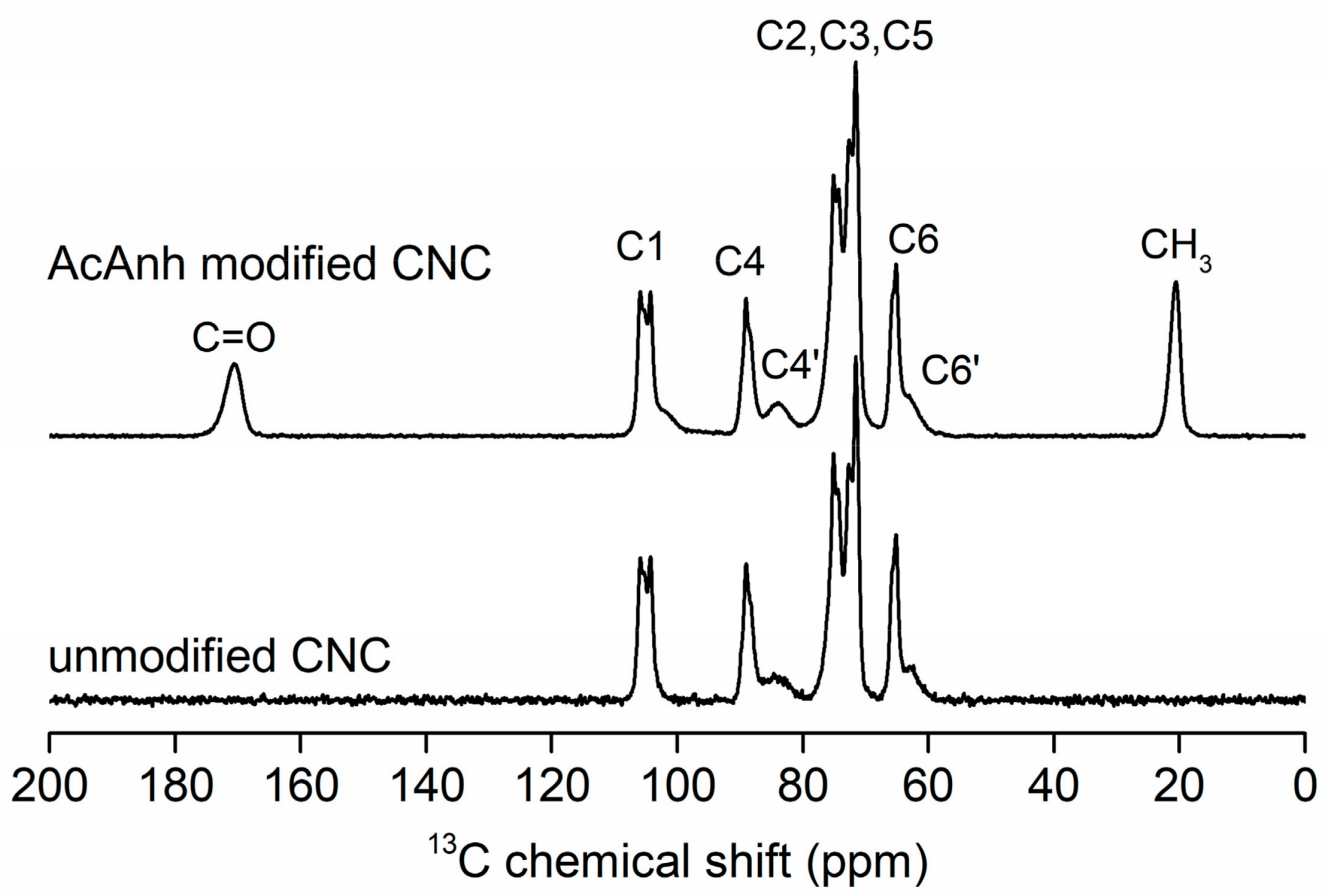
^13^C cross polarization-magic angle spinning (CP-MAS) NMR spectra of unmodified CNCs and CNCs modified with AcAnh.

The ^13^C cross polarization-magic angle spinning (CP-MAS) NMR spectrum of the unmodified CNCs shows narrow signals corresponding to the cellulose C1–C6 carbons in the crystalline region, while two low intensity and broad signals belong to the C4′ and C6′ carbons in the amorphous region (Figure 6) [38]. After CNC modification with AcAnh, two additional signals appeared at 170.8 ppm (C=O) and 20.8 ppm (CH_3_), which indicated successful esterification of CNCs (Figure 6). The degree of substitution (*DS*), calculated according to Equation (1), was found to be 0.56 ± 0.02. It is worth noting that this is an average value, meaning that the *DS* of the cellulose chains on the CNC surface could be higher than that of the chains in the bulk.

**Figure 7:**
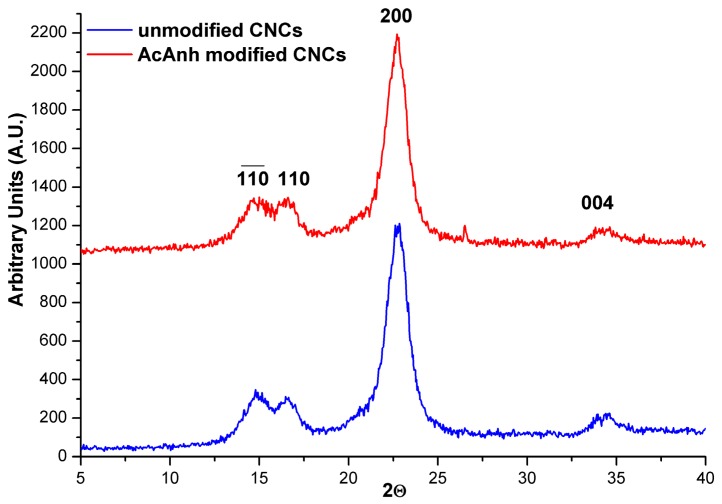
XRD diffractograms of neat CNCs (blue) and CNCs modified with AcAnh (red).

The impact of the esterification reaction and subsequent cleaning process on the CNC crystalline structure was studied by XRD powder diffraction. XRD diffractograms of the neat CNCs and with AcAnh-modified CNCs are shown in Figure 7. From the XRD patterns, we calculated the degrees of crystallinity of the samples using the simple and widely used Segal method [39]. The degrees of crystallinity of the neat CNCs and the CNCs modified with AcAnh are 88% and 86%, respectively, indicating only slightly disrupted CNC crystal structure during surface modification.

### 2.2. Nanocomposites of LLDPE and CNC

Nanocomposites were prepared from the LLDPE matrix and CNCs—either the unmodified CNCs or the CNCs modified with AcAnh—by extrusion at 160 °C and injection molding at 70 °C. The results of testing the tensile properties of the prepared specimens show that both CNC types influenced predominantly the Young’s modulus and elongation at break, while, in most cases, the tensile strength remained unaffected or even slightly deteriorated (Table 1). The increase in the nanocomposites’ Young’s modulus (by ~20%) was expected, however, the increase in strain at break (up to 90%) was rather surprising. Such results reveal that the impact resistance of the nanocomposites might be enhanced as well [40]. Unfortunately, Charpy impact resistance experiments could not be performed due to the high toughness of the materials, which did not break during the preliminary Charpy impact tests using notched specimens. The Young’s modulus as a function of the CNC concentration showed the highest increase when the so-called critical percolation concentration (CPC) had been reached. CPC is a function of the aspect ratio of the CNC particles [41,42]. In our case, the CPC of CNC particles was around 4.7 wt %, considering that the densities of CNC and LLDPE are 1.59 g/cm^3^ and 0.939 g/cm^3^, respectively. Around this concentration, the nanocomposites’ Young’s modulus was increased, supporting the so-called percolation theory. According to the CPC theory, the critical concentration of the CNC particles is essential for the formation of a continuous percolating reinforcing network of partially aggregated CNC particles throughout the polymer matrix. As opposed to other nanofillers, the CNCs have a unique capability of forming a strong hydrogen-bonded network, since the surface of the CNC particles is covered by a large number of hydroxyl groups [41]. The percolating CNC network is efficiently formed when the nanocomposites are prepared from the solution, however, we expected that this does not apply to the nanocomposites prepared from the polymer melt due to the high melt viscosity. To confirm this assumption, the temperature of the mold was increased from 100 °C to 110 °C, and finally to 120 °C. Results of testing the tensile properties as a function of the mold temperature showed an even higher increase in the nanocomposites’ Young’s modulus (by 45%), while the increase in tensile strength was much lower (only by 4% maximum) (Table 2) [32]. The elongation at break also increased as compared to that of the reference sample, however, the observed influence of the higher molding temperature was rather small (Table 2). The measured melting temperatures (*T*_m_) and melting enthalpies (Δ*H*_m_) of the nanocomposites (Table 1) show that *T*_m_ remained almost unaffected, while Δ*H*_m_ decreased by increasing the CNC concentration, especially when the CNC particles modified with AcAnh had been applied (Table 1). These results reveal that the presence of CNCs reduced the degree of crystallinity of the PE matrix [31]. Such an effect is more pronounced when the CNCs modified with AcAnh were used instead of the unmodified CNCs, indicating the enhanced compatibility of modified CNCs with the LLDPE matrix. The reduced degree of LLDPE crystallinity deteriorated the stiffness (Young’s modulus), but increased the strain at break, which can be an explanation for the increased strain at break upon CNC addition (Table 1 and Table 2), and also suggests that the change in stiffness is a sum of two opposite effects, i.e., the reinforcing effect of CNCs, which increases the Young’s modulus, and the ‘plasticizing’ effect, which reduces the polymer crystallinity, and consequently, the nanocomposite stiffness [42,43]. When the molding temperature had been increased to 100 °C or even to 120 °C, and the time of melt solidification and LLDPE crystallization had been prolonged, the Δ*H*_m_ increased due to higher LLDPE crystallinity, which resulted in a higher Young’s modulus, and thus the obtained results support our assumption. In this way, the highest reinforcing effect of the CNCs on the Young’s modulus (stiffness) was achieved (~45%; Table 2).

**Figure 8:**
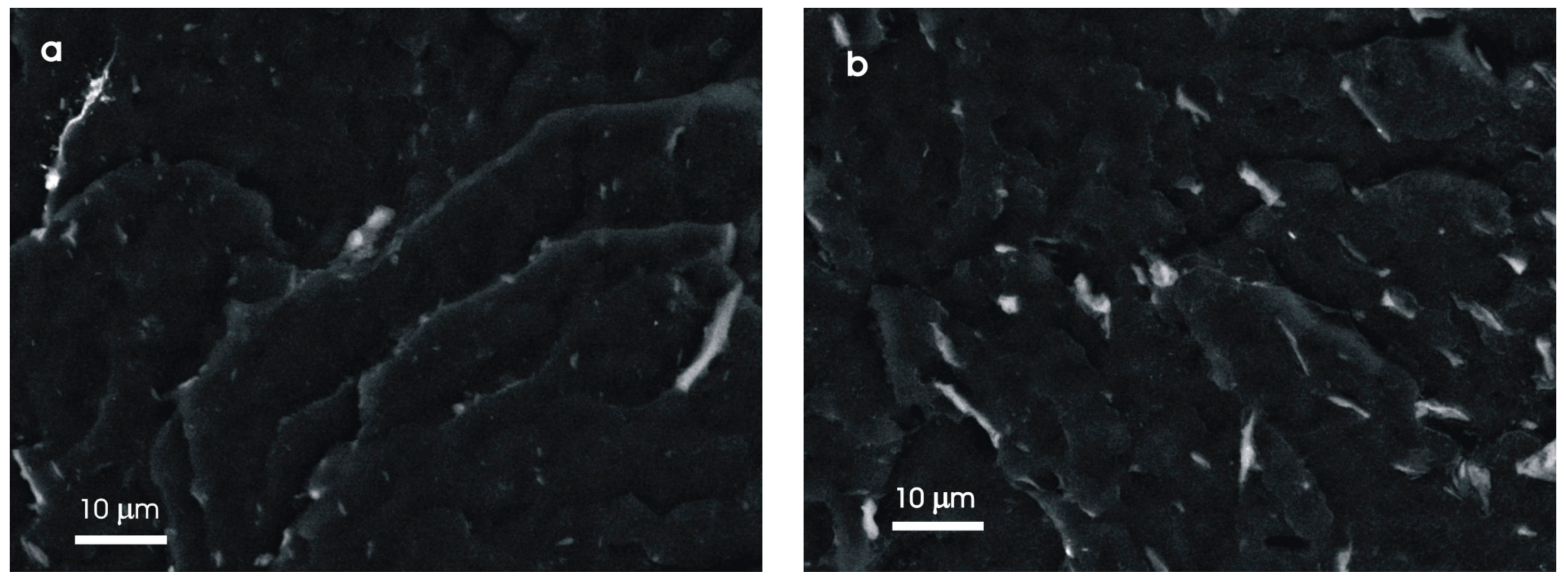

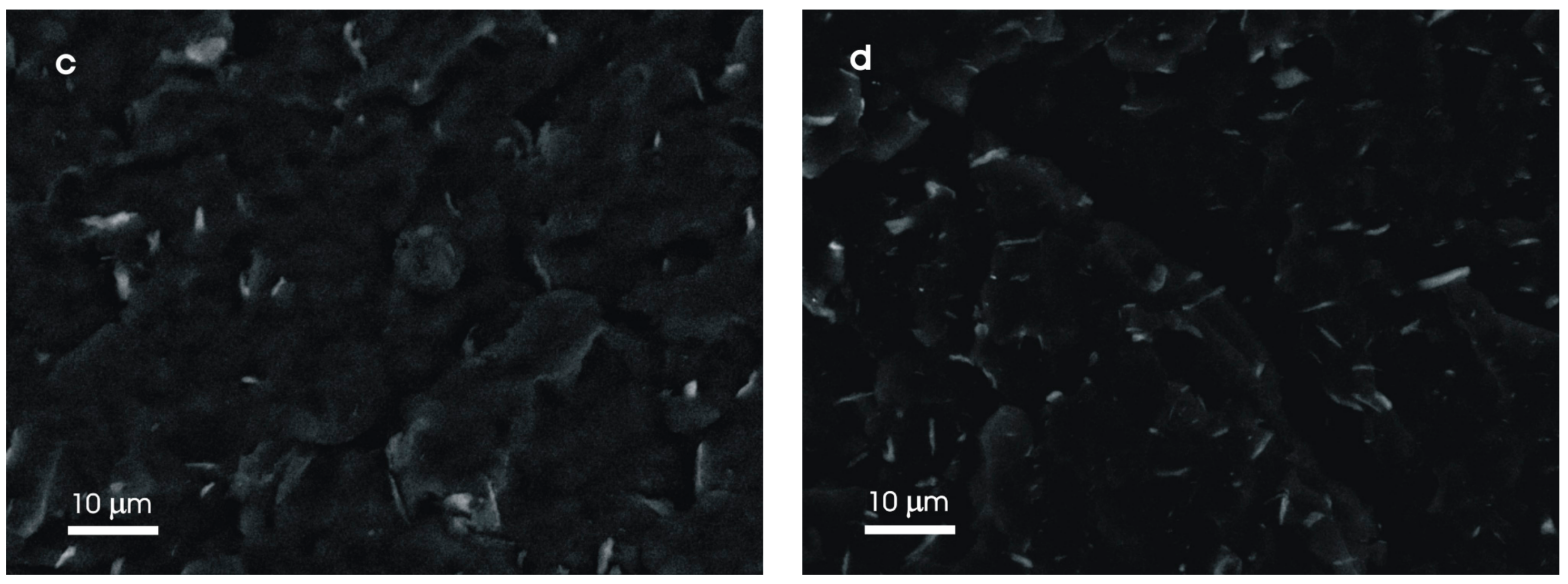
SEM micrographs (backs-scattered electrons) of CNC/LLDPE composites, showing CNC distribution in the LLDPE matrix as a function of CNC concentration and surface modification: (**a**) 2 wt % unmodified CNCs; (**b**) 5 wt % unmodified CNCs; (**c**) 2 wt % CNCs modified with AcAnh; (**d**) 5 wt % CNCs modified with AcAnh.

**Figure 9:**
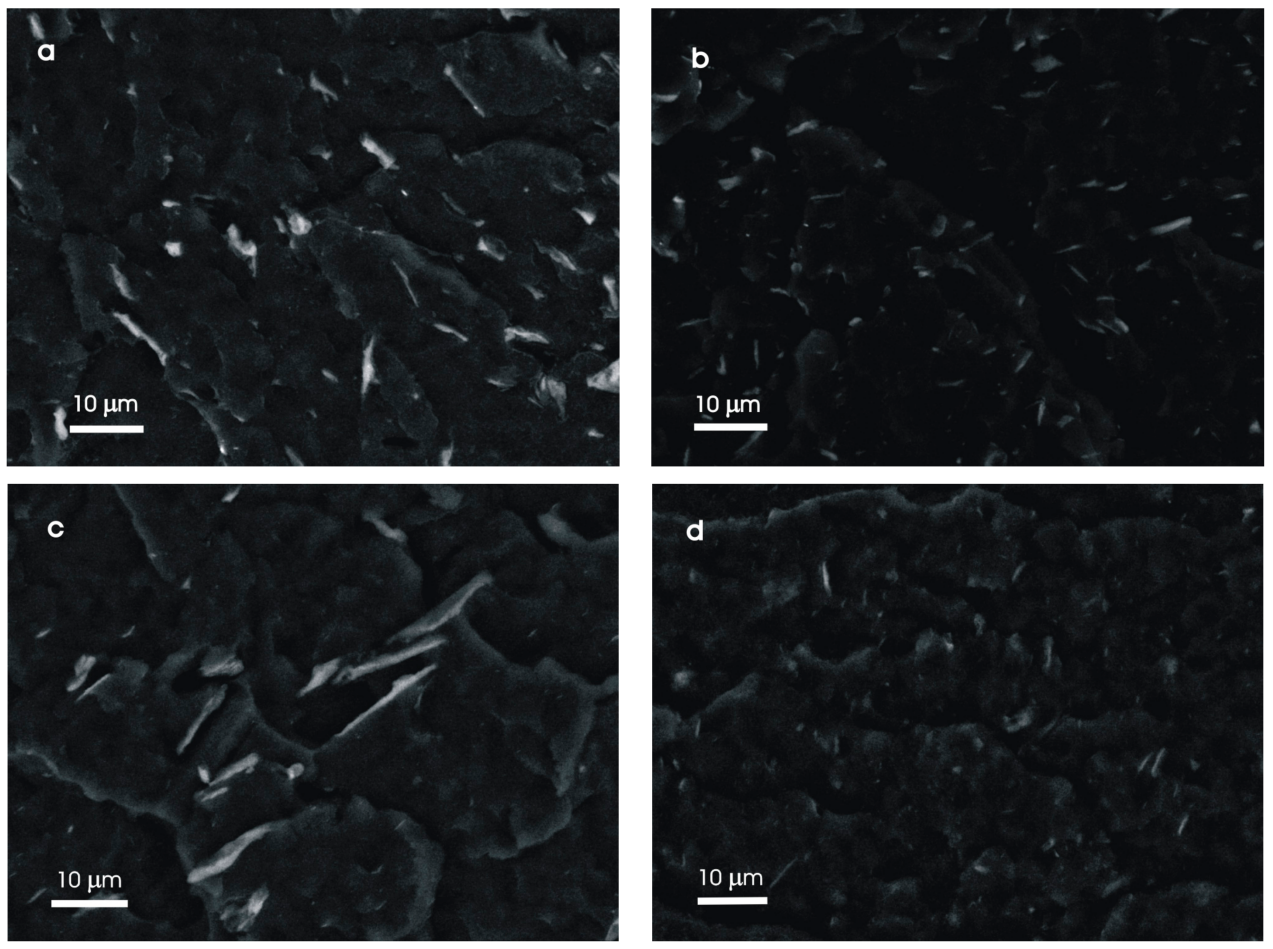
SEM micrographs (back-scattered electrons) of CNC/LLDPE composites, showing CNC distribution in the LLDPE matrix as a function of molding temperature and CNC surface modification: (**a**) 70 °C and unmodified CNCs; (**b**) 70 °C and CNCs modified with AcAnh; (**c**) 120 °C and unmodified CNCs; (**d**) 120 °C and CNCs modified with AcAnh. In all cases, the mass share of CNCs was 5 wt %.

The distribution of the CNC nanofiller in the PE matrix was studied by SEM microscopy of the fractures stained with I_2_ vapors using a back-scattered electron detector [42]. SEM micrographs in Figure 8 show the distributions of the unmodified and the AcAnh-modified CNCs in the PE matrix at the CNC concentrations of 2 wt % and 5 wt %. A comparison of the CNC distributions in both samples reveals that surface modification of CNCs with AcAnh enhanced the CNC compatibility with PE matrix. The average size of the CNC aggregated particles decreased from 2–20 μm in the PE/neat CNC composites to 0.5–5 μm in the PE/modified CNC composites. Unfortunately, the modification of CNCs by esterification did not reduce the size of the aggregates sufficiently to enable the formation of a CNC percolating network and to ensure improvement of the nanocomposite’s mechanical properties (Table 1). The only change observed for the composites of LLDPE and modified CNCs was a reduction in the strain at break. By increasing the molding temperature from 100 °C to 120 °C, a different influence was observed for the unmodified CNCs and the CNCs modified with AcAnh (Figure 9). For the PE/modified CNC composites, the size of the CNC aggregates remained mostly unchanged by increasing the molding temperature from 70 °C to 120 °C (Figure 9b,d), while for the PE/neat CNC composites, the size of the CNC aggregates significantly increased (Figure 9a,c). These results reveal enhanced aggregation of the unmodified CNC particles at higher molding temperatures due to the lower polymer melt viscosity. On the other hand, the CNCs modified with AcAnh showed no increase in the aggregates’ size, which is ascribed to enhanced compatibility of the modified CNCs with the PE matrix (Figure 9b,d). Unfortunately, the impact of the unmodified and modified CNCs on the mechanical properties (Table 1 and Table 2) was very similar, leading to a conclusion that CNC modification by esterification with AcAnh resulted in enhanced compatibility of the composite’s constituents, but it did not contribute to the improvement of the nanocomposite’s mechanical properties.

## 3. Materials and Methods

### 3.1. Materials and Procedures

#### 3.1.1. Materials

Acetic anhydride—AcAnh, 99%, Sigma Aldrich, St. Louis, MO, USA; butyric anhydride—ButyrAnh, 97%, Fluka, Buchs, Switzerland; hexanoic anhydride—HexAnh, 97%, Sigma Aldrich, St. Louis, MO, USA; valeric anhydride—ValerAnh, 97%, Sigma Aldrich, St. Louis, MO, USA; methacrylic anhydride—MethacrAnh, 94%, Sigma Aldrich, St. Louis, MO, USA; benzoic anhydride—BenzAnh, 98%, Fluka, Buchs Switzerland; 4-dimethylaminopyridine—DMAP, 99%, Sigma Aldrich, St. Louis, MO, USA; pyridine, Sigma Aldrich, St. Louis, MO, USA; 4-pyrrolidinopyridine—PyrolPyr, 98%, TCI, Tokyo, Japan; H_2_O_2_, 30%, Sigma Aldrich, St. Louis, MO, USA; triethylamine—TEA, 99%, Sigma Aldrich, St. Louis, MO, USA; tetrahydrofuran—THF, 99.5%, Merck, Darmstadt, Germany; acetone, 99.5%, Riedel-de-Haen, Seelze, Germany; Dioxane, 99.5%, Carlo Erba, Val de Reuil, France; poly(ethylene)-*graft*-maleic anhydride, 99%, Sigma Aldrich St. Louis, MO, USA; linear low-density polyethylene—LLDPE, REVOLVE N-307, Matrix Polymers, Northampton, UK.

#### 3.1.2. CNC Isolation

CNCs were isolated by the previously published ‘polyol method’ [36] from cotton linters in a mixture of diethylene glycol and glycerol (*w*/*w* = 70/30) at 150 °C and a 4 h reaction time using 3 wt % methanesulfonic acid as a catalyst. CNCs were washed 5× with dioxane, 3× with acetone, and finally with DI H_2_O. Each washing included centrifugation and sonification of CNCs. During the third and the fourth washing with dioxane, the CNCs were bleached with H_2_O_2_ overnight. Afterwards, the CNCs were suspended in DI H_2_O by sonication, frozen in liq. N_2_, and freeze dried for 72 h.

#### 3.1.3. CNC Modification with Anhydrides

The typical process of CNC modification with the AcAnh comprised weighing the freeze-dried CNCs, AcAnh, DMAP, and THF solvent in two equal portions of 4 g. In the first portion of THF, the CNCs (1 g) were suspended by sonication, while in the second portion of THF, the AcAnh (0.4 mol) and DMAP (3.2 mmol) were dissolved. The THF solution of AcAnh and DMAP was then added to the suspension of CNCs in THF. The whole system was additionally sonicated for 3 min, and afterwards, mixed with the magnetic stirrer for 24 h at room temperature. When CNCs were modified with other anhydrides, a longer reaction time (48 h or 72 h) was required. After reaction completion, the modified CNCs were washed 5× with acetone and 1× with DI H_2_O. The thus obtained products were kept in DI H_2_O until the preparation of nanocomposites.

#### 3.1.4. Preparation of Powder Mixtures of CNCs and LLDPE

The dry content of wet CNCs was determined prior to weighing the mixture components. The mass of the wet CNCs was calculated, taking into account the previously determined dry CNC content. Wet CNCs were weighed according to the dry CNC content of 0.25 g (or 0.1 g or 0.05 g), and afterwards, the CNCs were dispersed in 30 mL of DI H_2_O by sonication (2 min). Five grams of LLDPE powder were admixed to thus prepared CNC suspension. CNC/LLDPE mixture was sonicated for 5 min, frozen in liquid N_2_, and freeze dried.

#### 3.1.5. Melt Processing of CNC/LLDPE Mixtures

Prior to extrusion, 1.5 wt % of polyethylene-*graft*-maleic anhydride as a compatibilizer was added to the CNC/LLDPE mixture, and the components were mechanically mixed by shaking in order to homogenize them. The mixture was subsequently extruded with a Haake MiniLab extruder at 160 °C for 6 min at 50 rpm. The mixture was added to the extruder in two portions of 3 g. The extruded melt was ejected from the extruder at 100 rpm, and it was captured in a heated container (170 °C), which was further placed into a Haake Mini Jet molding machine to process the testing specimens by injection into a suitable mold heated to 70 °C at the pressure of 750 bar and time of 10 s, as well as the post pressure of 250 bar and time of 10 s. The molding temperature was varied from 100 °C, 110 °C, to 120 °C. Thus prepared specimens were used for the mechanical testing and other types of characterization; i.e., electron microscopy, differential scanning calorimetry (DSC), and dynamic mechanical analysis.

### 3.2. Characterization

Chemical composition of the unmodified and AcAnh-modified CNCs was studied by Fourier-transform infrared spectroscopy (FTIR) on an FTIR spectrometer Spectrum One (Perkin-Elmer, Waltham, MA, USA) in the transmittance mode, spectral range between 400 cm^−1^ and 4000 cm^−1^, and spectral resolution of 4 cm^−1^. Samples were prepared by a KBr pellet technique.

Solid-state NMR measurements were performed on a 600 MHz Varian NMR spectrometer (Varian, Palo Alto, CA, USA) using 1.6 mm HXY fast MAS probe. Samples were spun at 32 kHz. Protons were excited by the 90° pulse with a duration of 1.5 µs. The ^1^H–^13^C cross polarization magic-angle spinning (CP-MAS) experiment employed RAMP [44] during 5.0 ms CP block and high-power XiX heteronuclear decoupling during acquisition [45]. The numbers of scans were 2800, 5200, and 64,800 for the cellulose acetate, unmodified CNCs, and AcAnh-modified CNCs, respectively. A repetition delay of 1 s was used for both unmodified and modified CNCs, and a longer delay of 20 s was used for the cellulose acetate due to a slower ^1^H spin-lattice relaxation. The ^13^C Larmor frequency was 150.74 MHz, and the spectra were calibrated according to the ^13^C signal of the tetramethylsilane. The degree of substitution (*DS*) was calculated using the following equation:

*DS* = *A_CP_* × (*I*_*CH*3*CO*_/*I*_*C*1–*C*6_) (1)

where *I*_*CH*3*CO*_ is the sum of the areas of the carbon nuclei of ester groups, *I_C_*_1*–C*6_ is the integral area of the cellulose carbons (C1–C6), and *A_CP_* is a constant, which was calculated from the ratio of the integrals obtained from the ^13^C CP-MAS spectrum of the cellulose acetate with a known degree of substitution (*DS* = 2.4).

The size of CNC particles in DI H_2_O was measured by dynamic light-scattering (DLS) using a Malvern Zetasizer Nano-ZS (Malvern, Worchestershire, UK) at a scattering angle of 173°. The samples of CNCs were diluted with DI H_2_O and sonicated for 10 min. The CNC and H_2_O refractive indices of 1.478 and 1.3317, respectively, were used in calculations [46].

Unmodified and AcAnh-modified CNC samples were characterized by wide-angle X-ray diffraction using a Siemens D-5000 diffractometer (Siemens, Erlangen, Germany) with a Cu anode as an X-ray source. Diffractograms were measured at 25 °C in a 2Θ range from 5° to 40°, with a step of 0.04° and a step time of 340 s. Crystallinity index was calculated according to the widely used Segal method [39].

Tensile test experiments were performed according to the standard ISO 527 using a Shimadzu AGS-G Xplus dynamometer (Shimadzu, Kyoto, Japan) with an initial grip separation of 58 mm and a test speed from 0% to 0.25% = 2 mm/min and from 0.25% on = 200 mm/min using a pretension of 10 N. CPC was calculated according to the literature [42].

Differential scanning calorimetry (DSC) measurements were performed on a DSC-1 calorimeter (Mettler Toledo, Greifensee, Switzerland) in the temperature region from 25 °C to 200 °C. Two heating/cooling scans were performed with heating and cooling rates of 10 °C/min.

The morphology and size of the neat CNC particles, as well as the CNC distribution in the LLDPE matrix, were studied by scanning electron microscopy (SEM). The electron micrographs of the samples were taken on a Zeiss Supra 35 VP field emission electron microscope (Zeiss, Oberkochen, Germany) at an acceleration voltage of 20 kV using a secondary detector at a working distance of 4.85 mm for the neat CNCs, whereas the SEM micrographs of the nanocomposites’ cross-sections were taken at a 15 kV acceleration voltage using a back-scattered electron detector at a working distance of 8 mm. The CNC sample was dispersed in acetone by sonication, and a drop of dispersion was then transferred to a hot object glass, and then left to evaporate the solvent. The samples of molded CNC/LLDPE nanocomposites were broken in liquid N_2_ and placed on conductive sticky tape. The samples were stained with I_2_ vapors for 24 h and treated at 50 °C in a vacuum for 24 h to remove the unbound I_2_, and finally the samples were coated with C.

## 4. Conclusions

Cellulose nanocrystals (CNCs) were synthesized by the ‘polyol process’. The CNCs were further surface modified at room temperature in THF by various anhydrides, using DMAP as a catalyst. Among three catalysts tested, DMAP and PyrrolPyr proved to be highly effective, whereas pyridine was least effective. Because of DMAP’s low price, it was selected for the upscaled reaction to produce the modified CNCs in gram quantities. The reaction time for successful esterification of CNCs depended on the length of the anhydride side chain, as indicated by FTIR.

Unmodified and acetic anhydride-modified CNC particles were applied as the reinforcing nanofillers for the LLDPE. Nanocomposites were prepared by melt processing; that is, by extrusion at 160 °C for 10 min and injection molding, with the molding temperature being varied from 70 °C to 120 °C. The average size and size distribution of the CNC particles and their aggregates in the LLDPE matrix were studied by SEM, which revealed smaller sized aggregates of the modified CNCs, as compared to those of the unmodified CNCs, indicating enhanced compatibility of the modified CNCs with the PE matrix. The results of tensile testing of nanocomposites showed an increase in Young’s modulus of 20% and elongation at break of up to 90% when the CNC concentration had reached 5 wt %, which is close to the calculated critical percolation concentration. By increasing the molding temperature to 120 °C, the Young’s modulus further increased by ~45% due to the higher degree of crystallinity of the LLDPE matrix, indicating the reinforcing effect of CNCs on the material’s stiffness, however, the tensile strength was more or less unaffected. Despite improved compatibility between the modified CNCs and PE matrix, no improvement in the mechanical properties of the nanocomposites was observed, as compared to the properties of the nanocomposites of LLDPE and unmodified CNCs.

## Figures and Tables

**Table 1:** Mechanical and thermal properties of CNC/LLDPE (linear low-density poly(ethylene)) nanocomposites as a function of CNC weight fraction and surface modification.

Sample Designation	Surface Modification	CNC Fraction	Young’s Modulus	Tensile Strength	Strain at Break	*T*_m_	Δ*H*_m_
		wt %	MPa	MPa	%	°C	J g^−1^
NComp-2	-	2%	0.45 ± 0.02	19.1 ± 0.22	59.6 ± 9.3	129.3	135.4
NComp-3	-	5%	0.51 ± 0.08	21.5 ± 0.27	54.9 ± 8.9	128.7	132.2
NComp-4	AcAnh	1%	0.45 ± 0.04	20.7 ± 0.12	35.1 ± 5.8	129.5	122.5
NComp-5	AcAnh	2%	0.43 ± 0.03	20.9 ± 0.27	37.3 ± 4.0	128.5	121.8
NComp-6	AcAnh	5%	0.51 ± 0.08	21.3 ± 0.41	33.7 ± 6.1	129.4	121.2

**Table 2:** Mechanical and thermal properties of CNC/LLDPE nanocomposites as a function of molding temperature and CNC surface modification. In all cases, the mass share of CNCs was 5 wt %.

Sample Designation	Surface Modification	Molding Temp.	Young’s Modulus	Tensile Strength	Strain at Break	*T*_m_	Δ*H*_m_
		°C	MPa	MPa	%	°C	J g^−1^
NComp-7	-	100	0.53 ± 0.06	20.2 ± 0.21	59.9 ± 7.8	128.4	136.2
NComp-8	-	110	0.56 ± 0.03	20.6 ± 0.48	65.1 ± 7.3	128.6	136.4
NComp-9	-	120	0.62 ± 0.03	21.3 ± 0.01	57.7 ± 8.8	131.0	134.9
NComp-10	AcAnh	100	0.44 ± 0.09	19.2 ± 0.48	75.3 ± 9.5	128.5	134.4
NComp-11	AcAnh	110	0.59 ± 0.02	20.8 ± 0.36	50.2 ± 6.2	128.7	135.4
NComp-12	AcAnh	120	0.63 ± 0.04	20.9 ± 0.77	50.6 ± 8.9	130.2	134.0
